# Tapering or Discontinuation of Methotrexate in Stable Disease- Opinions From Rheumatoid Arthritis Patients and Rheumatology Providers at Two Academic Healthcare Centers

**DOI:** 10.21203/rs.3.rs-9142998/v1

**Published:** 2026-04-06

**Authors:** Peri Newman, Tarun Sharma, Rayford June, Nicole Wilson, Glennys Smith, Erik Lehman, Vandana Rai, Nancy Olsen, Sharon Banks

**Affiliations:** Penn State Milton S. Hershey Medical Center; Allegheny Health Network; Penn State Milton S. Hershey Medical Center; Allegheny Health Network; Allegheny Health Network; Penn State Milton S. Hershey Medical Center; Allegheny Health Network; Penn State Milton S. Hershey Medical Center; Penn State Milton S. Hershey Medical Center

**Keywords:** rheumatoid arthritis, methotrexate, medication tapering, patient preference, provider preference

## Abstract

**Background:**

Current treatment guidelines for rheumatoid arthritis (RA) favor discontinuation of methotrexate (MTX) over biologic (b) DMARDs. To assess acceptability of this approach, we conducted a cross-sectional study evaluating the perspectives of patients and providers on MTX tapering at one urban and one rural health care center.

**Methods:**

Rheumatologists and patients were surveyed at the two health centers, one in an urban location and one with a predominantly rural service area. Patient surveys collected demographics, history of MTX use, and views on tapering. Provider surveys included questions about years of rheumatology practice and strategies being employed for tapering. Data were analyzed using chi-square and Wilcoxon rank sum tests in both questionnaire groups.

**Results:**

Surveys were collected over a 3 year period, from January 2020 through the end of 2022. Of the 143 patient respondents, 78% female and 86% were White. Median duration of RA was 12 years. Tapering trials of MTX were more frequent at the urban facility (p = 0.02) and were more often considered in males with stable disease activity (p = 0.005, and p = 0.042,) and those with longer disease duration. Tapering concerns were greater for female patients on MTX for at least 8 years (p = 0.046). Surveys collected from 16 urban providers and 8 rural providers showed that those with less than 15 years of experience were more likely to consider tapering if patients were concurrently on other treatments favorable for tapering (p = 0.019) and were likely to consider patient preferences against tapering (p = 0.004). None of the other differences between the two centers were significant.

**Conclusion:**

We identified factors influencing tapering decisions for both patients and rheumatology providers, including age, race, sex, RA duration and provider’s years of experience. Consideration of these will facilitate designing acceptable future approaches to MTX tapering in patients with stable RA.

## Background

Rheumatoid arthritis (RA), a chronic autoimmune disease, affects 0.25–1% of the global population and leads to progressive joint damage and disability, particularly in adults over the age of 40.^1^ Prompt initiation of disease-modifying, anti-rheumatic drug (DMARD) therapy within three months of diagnosis decreases structural damage and prevents disease progression.^2^ The 2021 American College of Rheumatology Guidelines for the Treatment of Rheumatoid Arthritis recommend initial treatment with methotrexate (MTX) monotherapy for DMARD-naïve RA patients with moderate to severe disease activity.^3^ However, long-term immunosuppression and MTX use can lead to severe adverse reactions including serious infection and hepatotoxicity, especially in older patients with comorbidities.^4, 5^ Therefore, once RA patients achieve low disease activity or remission, MTX tapering should be considered. Successful drug tapering is achieved through shared decision making between patients and practitioners. Here, we surveyed RA patients and providers to determine factors influencing MTX tapering decisions.

Recent data from the ARCTIC REWIND trial suggest the possibility of achieving flare-free tapering of conventional synthetic DMARDs (csDMARDs) such as MTX in > 50% patients.^6^ While exploring biological predictors of successful tapering is important, it is equally important to understand patient and provider thoughts and preferences to help guide the tapering process.^7^ A previous interview-based study found RA patients’ and rheumatologists’ perspectives on tapering DMARDs vary and evolve over time, with rheumatologists generally open to tapering (not stopping) only when requested by their patients in some cases.^8^ Another study surveyed patients with stable RA and rheumatology providers and found patients were more likely to consider tapering bDMARDs and targeted synthetic (ts) DMARDs, especially when not being treated with concomitant csDMARDs or glucocorticoids.^9^ However, there is currently a lack of literature on patient and provider preferences within different demographics, including time in practice and duration of disease, that affect decisions to taper DMARDs.

In this study, we surveyed RA patients and providers practicing at two tertiary care academic centers to determine variables influencing MTX tapering decisions. Our study utilized differences in the two care centers to determine patient characteristics associated with MTX tapering decisions over a larger geographic population. We also evaluated the impact of the geographic distribution of RA patients (urban vs. rural setting) on tapering concerns and consideration.

## Methods

Patients and providers from Penn State Health (PSH) and Allegheny Health Network (AHN) were surveyed to analyze perspectives of MTX tapering between 01/01/2020 and 12/31/2022. Using electronic medical records from PSH and AHN, we identified patients diagnosed with RA via ICD9 or ICD-10 codes. We included RA patients ≥ 18 years old on a stable MTX dose for ≥ 6 months and attended an outpatient rheumatology clinic appointment between 1/1/2020 and 12/31/2022 at PSH or AHN. Exclusion criteria included absence of electronic health records and inability to complete a questionnaire because of cognitive, language, or literacy barriers. PSH patients represented a largely rural patient population, and AHN patients represented an urban population. Rheumatology providers practicing at PSH and AHN, including faculty, fellow trainees, and advanced practice providers were also included. Participation was voluntary.

Patients who met criteria and were seen by their provider were asked at the visit to complete the survey either electronically online into REDCap or in person at the visit. Providers were invited via email with a survey link to complete the survey electronically online into REDCap. The surveys were developed for this study and are included in the supplementary files (Supplementary File-1 Patient Methotrexate Tapering Survey and Supplementary File-2 Provider Methotrexate Taper Survey). Patient surveys collected respondents’ age, gender, race, ethnicity, RA disease history, self-assessed RA status (0 = very well, 10 = poor), duration of RA diagnosis and MTX use in years, and views on MTX tapering. Provider surveys asked number of years in practice as a rheumatologist (< 5 years, 5–15 years, and > 15 years), opinions on tapering patients with stable RA, and preferred tapering strategies. Providers at different experience levels were also asked to rate the importance of variables categorized as being in favor of or against MTX tapering.

This study was approved by the Institutional Review Boards of the Pennsylvania State University and the Allegheny Health Network and all patients provided written informed consent before participating. The study was performed in accordance with the Declaration of Helsinki.

Data were collected in one REDCap database. Patient data were stratified by healthcare network location, self-identified gender, self-identified race (White or non-White), and self-identified ethnicity. Descriptive statistics were completed for age, duration of MTX use, and RA duration. Provider data were stratified by site and years in practice.

Chi-square analysis compared survey responses between AHN and PSH patients and between male and female patients. Two-sample t-tests and Wilcoxon rank sum tests compared patient age, years with RA, and years on MTX between patients who responded positively or negatively to survey questions. Factor importance ratings were compared based on the number of years providers have practiced rheumatology using Wilcoxon rank sum tests. SAS (SAS Institute, Cary, NC) was used for all analyses, and statistical significance was set at p < 0.05.

## Results

### Patient perspectives

A total of 143 patients completed the survey (73 AHN; 70 PSH). The patient population was 78% female (n = 112) and 85% White (n = 121). Mean RA duration was 16.06 years (± 13.29 standard deviation [SD]). Self-assessed RA status (0 = very well, and 10 = poor) was significantly better in White patients (mean score = 5.06) than in non-White patients (mean score = 3.41; *p* = 0.007). AHN had significantly more non-White patients than PSH (*p* = 0.002, Table 1). PSH patients were older (≥ 65 old) than AHN patients (*p* = 0.013). Self-assessed RA status did not differ by site (p = 0.311)

MTX tapering consideration and patient concerns significantly differed by patient gender, study site, duration of RA, and duration of MTX (Table 2). When comparing tapering between study sites, more patients at AHN underwent MTX tapering than at PSH (*p* = 0.02). Across both AHN and PSH, male patients (n = 13 [41.9%]) underwent MTX tapering more frequently than female patients (n = 20 [17.9%], *p* = 0.005). MTX tapering was also considered more often by male patients (n = 29 [96.7%]) than female patients (n = 85 [81%], *p* = 0.042) and by patients diagnosed with RA > 21 years (*p* = 0.01). Patients treated with MTX ≥ 8 years were more likely to have previously tapered or discontinued MTX (p = 0.044) but also reported greater concern about risks of reducing or stopping the drug (p = 0.004), particularly related to flares (p = 0.002) and worsening disease (p = 0.001). Female patients were also more concerned about risks of flares than male patients (*p* = 0.046).

### Provider perspectives

Twenty-four providers (16 AHN; 8 PSH) completed the survey with a100% response rate. Ten providers (42%) had been practicing rheumatology for ≥ 15 years (Table 3). Variables in favor of or against tapering were ranked by importance, from 1 (very important) to 5 (least important). When comparing rankings between AHN and PSH providers, we found no significant differences (not shown). Comparing the group of providers with < 15 years of experience (n = 14) to those with greater experience, significant differences were found for two variables—concurrent DMARD therapy and patient preference (Table 3). Of the five variables in favor of tapering, only concurrent DMARD was considered significantly more important by providers who had been practicing for longer (*p* = 0.019). The importance of achieving remission, risks of long-term medication use, and patient noncompliance with monitoring did not significantly differ between providers. For the six variables against tapering, providers with more experience viewed patient preference as significantly less important than those with less experience (*p* = 0.004). Importance ratings for radiographic progression, multiple treatment failures, and challenges regaining remission after flare did not differ significantly.

## Discussion

In this study, surveys of RA patients and providers in two academic centers about MTX tapering found MTX tapering was more often attempted in male patients, patients treated at AHN, and patients with stable disease activity, long-term disease (> 6 years), and > 8 years of MTX use. Female patients with well-controlled RA were less likely to consider tapering and more concerned about the associated risks than their male counterparts. Greater concern of worsening disease and risk of flare was also observed in patients with > 8 years of MTX use. Furthermore, older patients were more likely to adhere to prescribed regimens. We also found rheumatology providers with < 15 years of experience were more likely to suggest MTX tapering to patients on concurrent DMARD therapy and considered patient preference less important than providers with more experience.

Previous studies on RA patient preferences report patients and providers to have concerns regarding increased risk of flares during DMARD tapering. For example, one study found patients were receptive to recommendations of medication dose reduction but expressed concerns about flare risks with tapering and the need for timely provider communication and a clear plan if tapering was unsuccessful.^10^ Another study investigating patient and rheumatologist perspectives on tapering DMARDs in RA found while acknowledging the potential benefits of tapering such as reducing side effects, both the patients and physicians interviewed in the study were cautious, noting lack of evidence.^8^ Fear of flare and time it would take to return to a stable low activity state remains an obstacle for patients and providers in DMARD tapering decisions.^8^ In our study, RA patients that had been treated with MTX > 8 years were more likely to be concerned about the risk of flares and worsening disease. Additionally, male patients were more likely to consider MTX tapering as female patients were more concerned about the risks associated with tapering. The AHN team has previously reported on the risk of flare in RA patients who had well-controlled disease.^10^ In this real-world cohort comparing tapering of different medications, flare rates were lowest while tapering in those on conventional synthetic DMARDs, such as MTX, compared to bDMARDs.^11^

The official guidelines for RA treatment recommend gradual discontinuation of MTX under certain conditions and with little evidence.^3^ Rheumatologists may be more likely to consider tapering or discontinuing DMARD therapy in their stable patients, but there are currently no strong guides for the best timing or strategies for tapering, and treatment is individualized to each patient and circumstance. This lack of strong guidelines leads to variation in provider preferences and decision making when considering MTX tapering. We found few differences in the importance ratings of variables influencing MTX tapering decisions between providers at AHN and PSH and those with different experience levels. However, providers with less experience (< 15 years practicing rheumatology) were more likely to suggest MTX tapering to patients on concurrent DMARD therapy and considered patient preference less important than providers with more experience.

This study has several strengths and limitations. Strengths include the inclusion of patients in both rural and urban settings for a broader view of MTX tapering in patients from different geographic locations. Providers with different backgrounds/education (advanced practice providers vs. medical physicians) and years in practice were included. Pre-determined standardized responses were used in the surveys to make data collection and interpretation more uniform. Notably, data collection occurred during the COVID-19 pandemic for which heightened awareness of the effect of medications on disease and health could have raised patient awareness about tapering issues. Limitations include a lack of patient diversity with most patient respondents being White, selection bias in the clinics chosen to participate in the survey, and recall bias on behalf of the patient in choosing how to answer the questions about their years on MTX and years of diagnosis. Finally, since we did not collect the number of total patients asked to complete the survey vs. the number collected; an accurate survey response rate could not be determined.

## Conclusions

Patients generally do not wish to take more medication than needed, and patient preferences on medication tapering or discontinuation in stable disease are important for shared decision making. Some of the results in our study are not unexpected, but pursuing guided strategies that are likely to succeed in medication dose reduction will require further study. Studies including patient and provider preferences on taper, especially studies such as this that build upon prior studies and validate prior thoughts and findings are needed. Furthermore, these results strongly suggest shared decision making among patients and providers to taper or discontinue medications including MTX will be critical to successful outcomes and provide a good starting point for future designed patient care standardized tapering guidelines.

## Supplementary Material

This is a list of supplementary files associated with this preprint. Click to download.

patientsurvey.pdf

ProviderMethotrexateTaperSurve.pdf

## Figures and Tables

**Table 1:** Comparison of self-reported demographics from 73 patients at the urban Allegheny Health Network (AHN) and 70 patients at the rural Penn State Health (PSH) sites†

Gender	Urban AHN (N = 73) n (%)	Rural PSH (N = 70) n (%)	*p*-value
		
Male	18 (24.7)	13 (18.6)	0.377
Female	55 (75.3)	57 (81.4)	
Race			
White	55 (77.5)	66 (95.7)	0.002*
Non-White	16 (22.5)	3 (4.3)	
Ethnicity			
Hispanic	0	3 (4.3)	0.113
Non-Hispanic	72 (100)	66 (96.7)	
Age			
Younger than 65	42 (61.8)	28 (40.6)	0.013*
At least 65	26 (38.2)	41 (59.4)	

* statistically significant

† total values do not equal 100% due to separate site enrollment

**Table 2:** Survey results to six questions asked of patients as categorized by institute, sex, age, and rheumatoid arthritis (RA) history

1. Consideration of MTX tapering if RA was well-controlled	Yes n (%)	*p*-value*	Yes (Mean ± SD)	No (Mean ± SD)	*p*-value**
				
Institute					
Penn State Health (rural)	55 (79.7)	0.121			
Allegheny Health Network (urban)	59 (89.4)				
Sex					
Male	29 (96.7)	**0.042**			
Female	85 (81.0)				
Age (years)			62.2 ± 13.5	63.8 ± 12.7	0.870
Years on MTX			9.8 ± 9.2	10.1 ± 7.1	0.481
Years with RA			16.3 ± 13.2	13.3 ± 10.6	0.607
2.Tapered off MTX in clinical practice due to stable disease activity					
Institute					
Penn State Health (rural)	11 (15.1)	**0.020**			
Allegheny Health Network (urban)	22 (31.4)				
Sex					
Male	13 (41.9)	**0.005**			
Female	20 (17.9)				
Age (years)			63.7 ± 11.6	62.3 ± 13.7	0.615
	Years on MTX			11.7 ± 7.2	9.5 ± 9.8	**0.015**
Years with RA			16.5 ± 11.5	15.9 ± 13.8	0.385
3. Concerned overall with reducing or stopping MTX					
Institute					
Penn State Health (rural)	27 (39.1)	0.675			
Allegheny Health Network (urban)	29 (42.7)				
Sex					
Male	10 (32.3)	0.267			
Female	46 (43.4)				
Age (years)			62.3 ± 13.1	62.2 ± 13.5	0.963
Years on MTX			11.5 ± 9.3	8.4 ± 8.4	**0.035**
Years with RA			17.9 ± 13.6	14.1 ± 12.1	0.061
4. Concerned about risk of flare when reducing or stopping MTX					
Institute					
Penn State Health (rural)	21 (30.0)	0.871			
Allegheny Health Network (urban)	21 (28.8)				
Sex					
Male	6 (19.4)	0.167			
Female	36 (32.1)				
Age (years)			60.03 ± 13.4	63.7 ± 13.1	0.136
	Years on MTX			12.2 ± 9.3	9.0 ± 9.2	**0.029**
Years with RA			19.2 ± 14.1	14.7 ± 12.8	**0.030**
5. Concerned about worsening when reducing or stopping MTX					
Institute					
Penn State Health (rural)	15 (21.4)	0.446			
Allegheny Health Network (urban)	12 (16.4)				
Sex					
Male	2 (6.5)	**0.046**			
Female	25 (22.3)				
Age (years)			60.9 ± 14.6	63.1 ± 12.9	0.441

Years on MTX			13.1 ± 9.1	9.2 ± 8.9	**0.034**
Years with RA			19.9 ± 14.7	15.2 ± 12.9	**0.046**
6. Skipped or forgotten to take MTX					
Institute					
Penn State Health (rural)	29 (41.4)	0.376			
Allegheny Health Network (urban)	25 (34.3)				
Sex					
Male	8 (25.8)	0.121			
Female	46 (41.1)				
Age (years)			57.1 ± 14.3	66.1 ± 11.4	**< 0.001**
Years on MTX			10.6 ± 8.3	9.6 ± 9.9	0.208
Years with RA			16.9 ± 12.2	15.6 ± 14.0	0.229

*Note*: SD =standard deviation

* *p*-value from chi-square test

** *p*-value from two-sample t-test (age) or Wilcoxon rank sum test (years on MTX, years with RA)

**Table 3:** Comparison of the mean importance ratings of the factors influencing providers’ tapering decision between with < 15 years in practice (YIP) and those with ≥ 15 YIP.

	< 15 YIP (N = 14)	≥ 15 YIP (N = 10)	
	Mean Importance Rating*		*p*-value
Variables in favor of tapering			
Stable remission	1.36	1.00	0.246
Risks of long-term MTX	2.57	3.10	0.319
Noncompliance with monitoring	1.93	2.00	0.685
Patient preference	2.21	1.90	0.547
On concurrent DMARD	1.93	1.10	**0.019**
Variables against tapering			
Risk of flare	1.29	1.30	0.756
Patient preference	1.65	2.60	**0.004**
Difficulty of recapturing remission	2.14	2.10	0.713
Failed multiple previous therapies	1.36	1.60	0.608
Risk of radiographic progression	2.29	1.06	0.166
MTX useful to reduce immunogenicity or boost effect of bDMARD	2.00	2.50	0.209

*Note*: bDMARD: biologic disease-modifying, anti-rheumatic drugs; DMARD: disease-modifying, anti-rheumatic drugs

* The importance rating scale ranged from 1 very important to 5 being least important. The mean value of the responses was calculated and presented here.

## Data Availability

Data have restricted access due to inclusion of protected health information but are available upon request to the corresponding author for an anonymized dataset.

## Abbreviations

RA: Rheumatoid Arthritis
DMARD: Disease Modifying Anti Rheumatic Drug
MTX: Methotrexate
csDMARD: conventional synthetic Disease Modifying Anti Rheumatic Drug
bDMARD: biologic Disease Modifying Anti Rheumatic Drug
tsDMARD: targeted synthetic Disease Modifying Anti Rheumatic Drug
PSH: Penn State Health
AHN: Allegheny Health Network
